# Identification and validation of hub genes in drug induced acute kidney injury basing on integrated transcriptomic analysis

**DOI:** 10.3389/fimmu.2023.1126348

**Published:** 2023-03-29

**Authors:** Yi-Xuan Deng, Kun Liu, Qun-Xiang Qiu, Zhi-Yao Tang, Rui-Man Que, Dian-Ke Li, Xu-Rui Gu, Guang-Liang Zhou, Yi-Feng Wu, Ling-Yun Zhou, Wen-Jun Yin, Xiao-Cong Zuo

**Affiliations:** ^1^ Department of Pharmacy, The Third Xiangya Hospital, Central South University, Changsha, China; ^2^ Department of Hematology, The Third Affiliated Hospital of Chongqing Medical University, Chongqing, China

**Keywords:** drug-induced acute kidney injury, Limma package, hub genes, *in vitro* and *in vivo* validation, weighted gene co-expression network analysis

## Abstract

**Background:**

Drug-induced acute kidney damage (DI-AKI) is a clinical phenomenon of rapid loss of kidney function over a brief period of time as a consequence of the using of medicines. The lack of a specialized treatment and the instability of traditional kidney injury markers to detect DI-AKI frequently result in the development of chronic kidney disease. Thus, it is crucial to continue screening for DI-AKI hub genes and specific biomarkers.

**Methods:**

Differentially expressed genes (DEGs) of group iohexol, cisplatin, and vancomycin’s were analyzed using Limma package, and the intersection was calculated. DEGs were then put into String database to create a network of protein-protein interactions (PPI). Ten algorithms are used in the Cytohubba plugin to find the common hub genes. Three DI-AKI models’ hub gene expression was verified *in vivo* and *in vitro* using PCR and western blot. To investigate the hub gene’s potential as a biomarker, protein levels of mouse serum and urine were measured by ELISA kits. The UUO, IRI and aristolochic acid I-induced nephrotoxicity (AAN) datasets in the GEO database were utilized for external data verification by WGCNA and Limma package. Finally, the Elisa kit was used to identify DI-AKI patient samples.

**Results:**

95 up-regulated common DEGs and 32 down-regulated common DEGs were obtained using Limma package. A PPI network with 84 nodes and 24 edges was built with confidence >0.4. Four hub genes were obtained by Algorithms of Cytohubba plugin, including TLR4, AOC3, IRF4 and TNFAIP6. Then, we discovered that the protein and mRNA levels of four hub genes were significantly changed in the DI-AKI model *in vivo* and *in vitro*. External data validation revealed that only the AAN model, which also belonged to DI-AKI model, had significant difference in these hub genes, whereas IRI and UUO did not. Finally, we found that plasma TLR4 levels were higher in patients with DI-AKI, especially in vancomycin-induced AKI.

**Conclusion:**

The immune system and inflammation are key factors in DI-AKI. We discovered the immunological and inflammatory-related genes TLR4, AOC3, IRF4, and TNFAIP6, which may be promising specific biomarkers and essential hub genes for the prevention and identification of DI-AKI.

## 1 Introduction

Acute kidney injury (AKI) is a common disease affecting 15% of hospitalized patients and up to 40-60% of patients in intensive care units (1). Higher AKI severity is associated with increased mortality, with AKI accounting for 1.7 million deaths each year (2, 3). Medications are a relatively common cause of AKI, especially in hospitalized patients exposed to multiple medications (4). Study found that 71.6% of AKI patients had used nephrotoxic drugs prior to or during renal injury (5). Clinically, there are no specific and effective treatment measure for DI-AKI, especially the most common acute tubular injury. The treatments for DI-AKI are largely conservative, focusing on discontinuation of drug use to avoid further kidney damage (4). Therefore, The early identification and diagnosis of nephrotoxicity, as well as in-depth research on key genes and mechanisms are crucial for the prevention and treatment of DI-AKI are crucial for prevention (6).

Currently, clinical identification of medication nephrotoxicity is challenging, and there is an unmet need for biomarkers that can accurately identify DI-AKI (7). Histopathologic alterations can successfully detect early drug harm, but due to their intrusive character, they can only be employed in animals and cannot be applied to clinical patients (8). In clinical practice, creatinine is typically utilized as a biomarker of DI-AKI, yet creatinine has certain faults when used in DI-AKI (7). During the acute phase of DI-AKI, serum creatinine may not alter until a serious drug-induced damage or further injury takes place. Drug-induced hemodynamic alterations and effects on the tubular secretion of creatinine can both affect creatinine levels (9). Other widely used biomarkers, such as neutrophil gelatinase-associated lipocalin (NGAL), kidney injury molecule-1 (KIM-1), urinary tissue inhibitor of metalloproteinase 2 (TIMP-2) paired with urine insulin-like growth factor binding protein 7 (IGFBP-7) ([TIMP-2]·[IGFBP-7]), etc., are similarly insufficient for describing DI-AKI and vary amongst DI-AKI. In patients with contrast media (CM) or vancomycin-induced AKI, several investigations found that NGAL, KIM-1, and [TIMP-2]·[IGFBP-7] were not substantially changed (10–12). Therefore, relying on conventional kidney damage indicators to identify DI-AKI makes early diagnosis more difficult (13). In-depth exploration of new DI-AKI biomarkers is essential to promote early diagnosis and identification of DI-AKI in high-risk patients and reduce the clinical burden of this complication.

Different types of DI-AKI frequently result from the toxic side effects of medicines and their renal therapy. While different medications can harm any tubular segment, the tubules are the common target. Therefore, acute renal tubule injury is the basis of drug-induced AKI with varied mechanisms (14). It frequently occurs with the administration of many medications, including contrast media, antibacterial medicines, and chemotherapeutic drugs (4). The three prominent medications among them are vancomycin, cisplatin, and Iohexol. They are all first-line diagnosis and treatment drugs and are widely used in clinic. As one of the most effective chemotherapy drugs, cisplatin is widely used in the treatment of a variety of malignant diseases, including head and neck, testicular, bladder, and ovarian cancer (15). However, the use of cisplatin in clinical practice is limited by its side effects, especially nephrotoxicity. AKI has been reported in 20%-35% of patients after administration of cisplatin cisplatin (16). The concentration of cisplatin in PTECs is five time higher than that in blood due to the uptake and excretion of local proximal tubular transporters (16). Contrast medium represented by iohexol is routinely injected intravenously prior to CT angiography of the urinary tract, spinal cord, femoral joint, and lymphatic system (17). Contrase-induced AKI (CI-AKI) occurs in up to 30% of patients who receive CM (17, 18). In addition, CI-AKI and CKD are both associated with increased mortality (18, 19). Vancomycin is a widely used highly hydrophilic glycopeptide antibiotic that is considered the gold standard for the treatment of methicillin-resistant Staphylococcus aureus (MRSA) and methicillin-resistant Staphylococcus epidermidis infections (20). Its best bactericidal efficacy and relatively low price make it the most commonly used antibiotic, in up to 35% of hospitalized infected patients (14, 20). Vancomycin is associated with a higher risk of AKI than most other antibiotics, with an incidence estimated at 5-20% (14, 20).

To sum up, our objective is to identify potential common hub genes and biomarkers of DI-AKI using high-throughput sequencing results from the three major drugs represented by iohexol, cisplatin, and vancomycin, and to validate them using cell, animal models and public sequencing data, which may aid in the earlier and more accurate prevention and treatment of DI-AKI.

## 2 Materials and methods

### 2.1 Cell culture and microarray data collection

The HK-2 cells (Zhongqiaoxinzhou Biotechnology Co., Ltd., Shanghai, China) were cultured at 37°C in 5% CO2 in Dulbecco’s Modified Eagle’s Medium-F12 (DMEM/F12) containing 10% fetal bovine serum, streptomycin (100 g/mL), and penicillin (100 U/mL). After 48 hours of treatment with iohexol (75 mg I/mL), cisplatin (5 μM) or vancomycin (4 mM) separately, cells were harvested by using Trizol reagent. Then we sent the cells to Novogene for sequencing analysis. The gene expression matrices of the aristolochic acid I-induced nephrotoxicity (AAN), the renal ischemia-reperfusion injury (IRI), and the unilateral ureteral obstruction (UUO) were taken from the datasets GSE136276, GSE108195 and GSE156181, which included from Gene Expression Omnibus (GEO, https://www.ncbi.nlm.nih.gov/geo/) public database (21), and used as external validation data sets. More information was shown in **Table 1**.

**Table 1:** Information for selected microarray datasets.

GEO Accession Total samples	Selected samples Platform	Source tissue	Sample type
GSE136276 (10 Samples)	GPL23038	Kidney	5 WT_AAI 5 WT_Water
GSE108195 (4 Samples)	GPL7202	Kidney	2 WT 2 WT_IRI
GSE156181 (4 Samples)	GPL11202	Kidney	2 WT 2WT_UUO

### 2.2 Differentially expressed gene screening

Principal component analysis (PCA) was firstly carried out to Reveal the condition of the samples under different treatments. The Limma package of R software was then used to identify the differentially expressed genes (DEGs) between the control group and iohexol, the control group and cisplatin, or the control group and vancomycin. *P-Value <*0.05 and |log2 fold change (FC)| >1 was regarded as the threshold to determine the DEGs (22). Similarly, series matrix obtained from the GSE136276 were analyzed for DEGs using Limma following criteria *P-Value <*0.01 and |log2 fold change (FC)| >1.5. The packages ggplot2 and heatmap are used for visual presentation of those DEGs. The Limma package were also performed differential expression analysis in the ischemia reperfusion mouse model and the unilateral ureteral obstruction mouse model from GSE108195 and GSE156181 (*P-Value* < 0.05 and |log2 fold change (FC)| >1).

### 2.3 Intersection of DEGs and functional enrichment analysis

Compared with the control group, the DEGs that were co-up-regulated in the three groups were intersected by R, and the co-down-regulated were also performed in this way. Then, using the R package “clusterprofiler”, we performed Gene Ontology (GO) enrichment for biological process (BP), cellular component (CC), and molecular function (MF) to determine the functional roles of the co-upregulated or down-regulated DEGs. Kyoto Encyclopedia of Genes and Genomes (KEGG) was utilized to identify the enriched pathways of all DEGs.

### 2.4 Protein–protein interaction network construction

By uploading all DEGs to the STRING online database (23), the PPI network with 84 nodes and 25 edges with confidence greater than 0.4 was constructed. We then visualized the PPI network by importing the observations into Cytoscape software (24).

### 2.5 Hub genes identification

Cytohubba, as a Cytoscape plugin, is applied to search for the hub genes. To gather as many accurate hub genes as possible, 10 different algorithms in Cytohubba were applied, including MCC, DMNC, MNC, Degree, EPC, BottleNeck, EcCentricity, Closeness, Radiality, and Betweenness (each strategy may predict the hub genes). The top ten rated genes in each algorithm were considered the algorithm’s hub genes. After calculating, we intersected the majority of these methods, and lastly, four genes that appeared to identified as the most plausible hub genes. The results were shown using the R package “UpSetR,” with the hub genes indicated by the red bar (25).

### 2.6 Cell counting kit-8 assay

Cell viability was determined using the Cell Counting Kit 8 (CCK8, Apexbio, Houston, USA) according to the manufacturer’s procedure. Cells were seeded and cultured in 100 μL medium at a density of 1 × 10^3^/well into 96-well microplates (Corning, USA). The cells were next treated with different concentrations of iohexol (12.5, 25, 50, 75 and 100 mg I/mL), cisplatin (3, 5, 8, 10, 20 μM) or vancomycin (0.5, 1.0, 2.0, 4.0, 6.0 mM). After 48h of treatment, 10 μL of CCK‐8 reagent was added to each well and incubated for at least 1h. Absorbance was analyzed at 450nm using a microplate reader (Bio‐Rad, Hercules, CA, USA) using cell-free Wells as a blank. Cell viability is expressed as absorbance.

### 2.7 Establishment of AKI mouse models

Male C57BL/6J mice (6-8 weeks, 20-25 g) were purchased by the Department of Laboratory Animals of Central South University (Hunan, China). Iohexol-induced AKI mice were anesthetized by intraperitoneal injection of 1% pentobarbital sodium (80 mg/kg), and were performed right nephrectomy surgery. After three weeks, the mice were dehydrated for 48 hours, 10 mL/kg furosemide was injected intraperitoneally, and 30 min later, 15 mL/kg iohexol (350 mg I/mL, Shanghai GE Pharmaceutical Co., LTD.) was injected *via* tail vein. Mice were fed freely after iohexol treatment. 24 hours after modeling, the mice were sacrificed. Mice with cisplatin-induced AKI were given a single intraperitoneal injection of 20 mg/kg cisplatin (P4394, Sigma-Aldrich), allowed to eat freely before and after the injection, and then were euthanized 72 hours later. Vancomycin-induced AKI mice were intraperitoneally injected 600 mg/kg/day vancomycin (HY-17362, MedChemExpress) for 7 days, and fed freely before and after vancomycin treatment and 24 hours after modeling, the mice were sacrificed. All blood samples of mice were collected from orbit to detect serum creatinine and urea nitrogen levels, and kidney tissues were isolated for RNA extraction.

### 2.8 Measurement of the renal injury

The serum creatinine (Scr) and blood urea nitrogen (BUN) levels were measured by automatic biochemistry analyzer (Hitachi 7600A, Japan) to assess renal injury.

### 2.9 Histological evaluation

The kidneys were fixed in 10% formalin, decalcified, and embedded in paraffin. Sections (3μm) were deparaffined, rehydrated and subjected with hematoxylin eosin (HE). For a semiquantitative study of morphological alterations, 10 low-magnification (x200) and high-magnification (x400) regions of the kidneys were randomly chosen. Grade 0, normal; Grade 1, 0-10%; Grade 2, 11-25%; Grade 3, 26-45%; Grade 4, 46-75%; and Grade 5, 76-100% were used to grade the tubular damage based on the degree of loss of brush border, cast formation, foamy degeneration, and detachment of tubular cells on a semiquantitative scale.

### 2.10 Quantitative real time PCR assay

HK-2 cells were treated with iohexol (75 mg I/ml), cisplatin (5 μM) or vancomycin (4 mM) separately in 6-well cell culture plates for 48 hours. Then Total RNA was isolated from cultured cells using Trizol reagent (TransGen Biotech, Beijing, China). A total 1μg RNA was reverse transcribed into cDNA with the RT kit (TransGen Biotech, Beijing, China). Real time PCR was performed using SYBER green mixture kit with gene-specific primers (**Table 2**). β-actin was used as internal control to calculate the relative mRNA expression of hub genes.

**Table 2:** The primers used for PCR.

Gene	GenBank entry	Primer	Sequence (5′ →3′)
β-actin	NC_000007.14	HF HR	CCACCATGTACCCAGGCATT CGGACTCATCGTACTCCTGC
AOC3	NC_000017.11	HF HR	GCCAATCCCTAAGTGTGGCT AACAGAGAGCGAGGGGGTAT
IRF4	NC_000006.12	HF HR	TGCACCCAGATCCTATAGCC CTCCATCGTCATCATCATCG
TNFAIP6	NC_000002.12	HF HR	CCTGGCCACCTTCTTCTTGT TTGCATTTCTCGGAGCCTGT
TLR4	NC_000009.12	HF HR	GAGGAGATCGAGGCAGAAGC TGCATTCCGTTTTGGCAAGG
β-actin	NC_000071.7	MF MR	GAGTCTGAAGTCGGGACCAC TTTTCCTCTTGCCTCCTGAA
Aoc3	NC_000077.7	MF MR	CCGCATCCAGATACTCAGCTT CGTCACCCAGGCTACCAAG
Irf4	NC_000079.7	MF MR	AGTCACACCCAGAAATCCCATATC CAGACCTTATGCTTGGCTCAATG
Tnfaip6	NC_000068.8	MF MR	AGGCCGTATGTGAATTTGAAGGT TGCATGTGGGTTGTAGCAATAGG
Tlr4	NC_000070.7	MF MR	TCCCTGCATAGAGGTGTGAAATT CCACAGCCACCAGATTCTCTAAA

F, forward; R, reverse.

### 2.11 Western blot assay

The samples of HK-2 cells and mice were lysed in RIPA buffer supplemented with protease inhibitors. The Bradford method was then used to calculate the protein sample’s concentration. Protein (10~20 μg) was separated on 4%–12% SDS-PAGE and transferred onto PVDF membranes. The samples were blocked for 1 hour, then incubated at 4°C overnight with AOC3 (Affinity Biosciences, DF6745, 1:1000), TLR4 (Affinity Biosciences, AF7017, 1:1000), IRF4 (Abclonal, A0524, 1:1000), or TNFAIP6 (Affinity Biosciences, AF5492, 1:1000). Following a one-hour incubation period at room temperature with secondary antibodies, the blots signaling were then obtained with chemiluminescence agents, and measured with Image Lab software. Target protein concentrations were compared to the endogenous control (β-actin, Proteintech, 66009-1-Ig, 1:5000).

### 2.12 Weighted gene co-expression network analysis and module gene selection

R package WGCNA was used to construct a scale-free network based on gene expression of ANN mice. The top 5000 of the median absolute deviation (MAD) of genes was selected for further analysis. Then, the appropriate co-expression similarity-derived “soft” (β) was selected for computing adjacency. The topological overlap matrix (TOM) was further calculated by the adjacency. The modules were detected by hierarchical clustering and dynamic tree cutting function. The genes with the same expression profile were divided into gene modules by the average linkage hierarchical clustering method based on the TOM dissimilarity measure and the minimum genome size (n = 30). Finally, the dissimilarity of module eigengenes was calculated, a cut line for the module dendrogram was selected, and multiple modules were joined for further analysis. The module with the highest correlation with AKI was selected, and the genes contained in the module were intersected with the DEGs obtained by LIMMA package. The outcomes were visualized by R package anRichment and ggplot2.

### 2.13 Patients

21 human plasma samples were collected to further verify the hub gene in clinical practice. The definition of vancomycin-induced AKI and cisplatin-indued AKI were based on Kidney Disease: Improving Global Outcomes (KDIGO) criteria, using only serum creatinine criteria, increase in Scr by 50% within 7 days or increase in Scr by 0.3 mg/dL (26.5 μmol/L) within 2 days (2). The definition of iohexol-induced AKI was defined as an increase in serum creatinine level ≥ 25% or ≥0.5 mg/dL (≥44 μmol/L) from baseline at 3 to 5 days (26).

### 2.14 Enzyme-linked immunosorbent assay

Centrifuging blood samples at 1000g for 30min and storing the plasma at -80°C. Following the directions, ELISA kits (YJ027583, YJ111654, YJ769180, YJ308102, YJ037978; Shanghai Enzyme-linked Biotechnology Co., Ltd.) were used for quantification by ELISA. In order to match the concentration of the relevant proteins into the linear range of the standard curve, the blood samples were diluted at a ratio of 1:50 before to ELISA.

### 2.15 Immunohistochemistry

The slices were dewaxed in anhydrous ethanol and dewaxing solution. Then, the slices were incubated in EDTA at 120°C for 5 min, then at room temperature with 3% H_2_O_2_ and in the dark for 25 min. The tissue was sealed for 30 minutes at room temperature after being evenly coated with 3% BSA. The sections were treated with the primary antibody (TLR4, Affinity Biosciences, AF7017, 1:100), and then they were laid flat in a moist box at 4°C and incubated for an overnight period. The secondary antibody labeled by HRP was applied to cover the tissue after the sections were washed in PBS. It was then incubated at room temperature for 50 min. Following a PBS wash, a freshly produced DAB color developing solution was applied to regulate the color development time under a microscope. The nucleus was stained with hematoxylin again and then examined under an optical microscope after being dehydrated with an alcohol gradient and sealed with neutral glue.

### 2.16 Statistical analysis

All experimental results were presented as the mean ± SD. The two tailed Student’s t-test was used to analyze the differences between the two groups. One-way ANOVA with Tukey’s *post hoc* tests was performed for comparing differences between groups. Each assay condition was performed at least three times for all experimental assays. All data collected were statistically analyzed. The *P-Value* < 0.05 is considered statistically significant.

## 3 Results

### 3.1 Identification of differentially expressed genes

**Figure 1:**
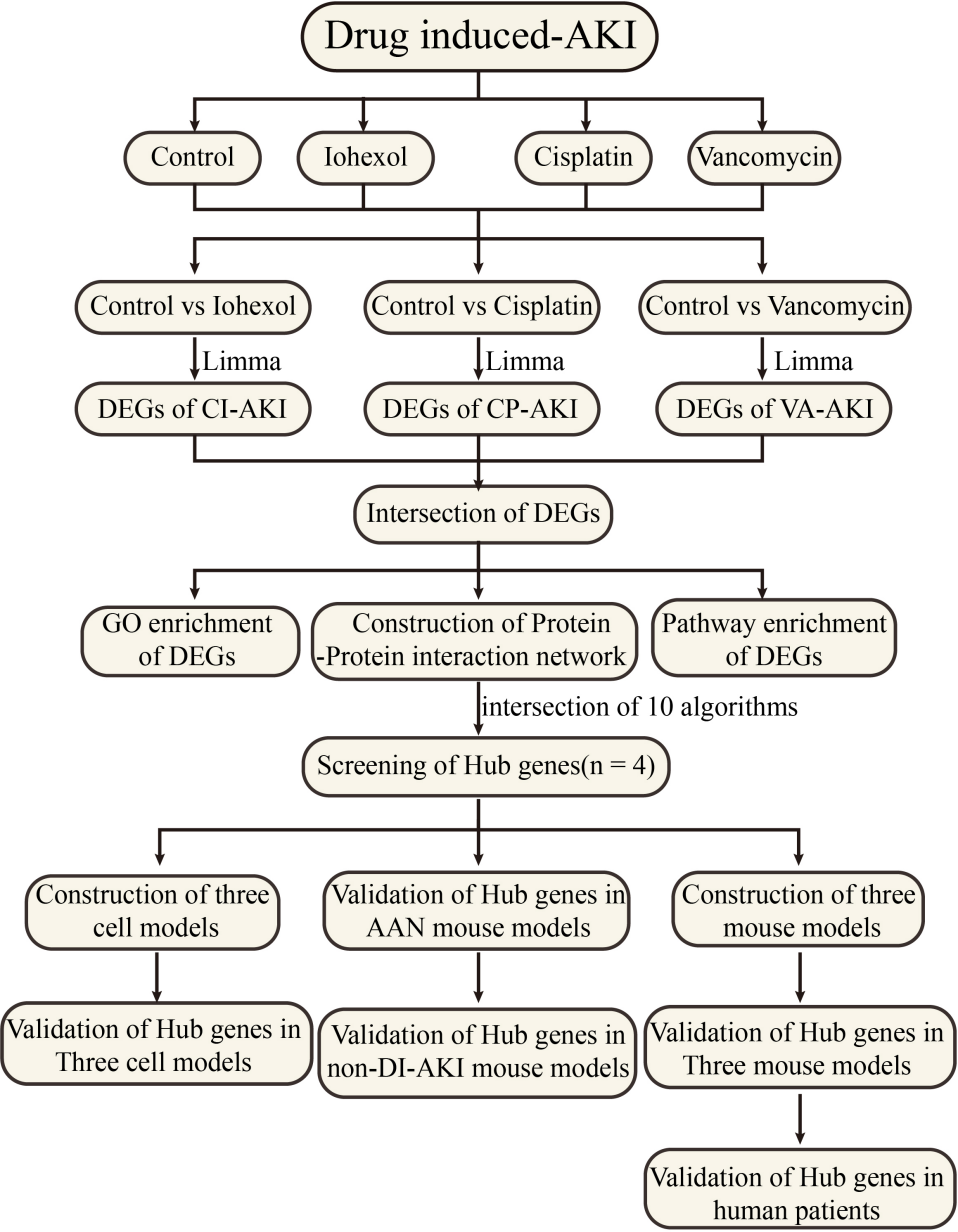
Work flowchart. Limma, linear models for microarray data; DEGs, differentially expressed genes; GSE, gene expression omnibus series.

As shown in **Figure 1**, mRNA sequencing technology was employed to determine alterations in mRNA expression of DI-AKI cell models treatment with iohexol, cisplatin and vancomycin. LIMMA was used for a thorough analysis to find differential genes. String database was used to predict protein-protein interactions, and then important hub genes were identified using ten algorithms in the cytohubba software. Following the discovery of the hub genes, the levels of their expression were confirmed in cell and mouse samples, including cellular mRNA and protein levels and mouse kidney mRNA and protein levels. Afterwards, ELISA kits were used to gauge the protein concentrations in mice’s serum and urine in order to investigate its potential as a biomarker. Date of the AAN, UUO and IRI models from public databases were validated to see if they were specific indicators. Finally, the protein levels of key hub genes were detected in the serum of patients with DI-AKI to finally determine their biomarker potential.

**Figure 2:**
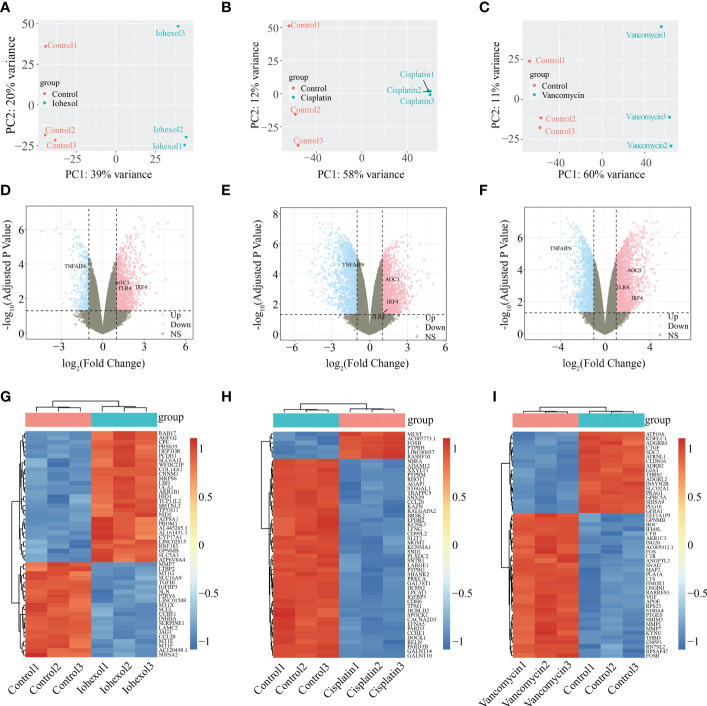
Identification of DEGs from three AKI datasets. **(A–C)** Differences in principal components between samples treated with iohexol, cisplatin, or vancomycin compared to controls. The control group is represented by red dots, whereas the treatment group is represented by blue dots. **(D–F)** Volcano plots of DEGs identified from the three datasets. Pink dots indicates up-regulated DEGs and the blue indicates down-regulated DEGs. **(G–I)** Heatmap of top 50 DEGs rated by *P-Value* identified in three AKI datasets respectively. AKI, acute kidney injury; DEGs, differentially expressed genes.

According to PCA alg orithm, the principal component differences between samples under different treatments were obtained. Groups after treatment with three different drugs respectively were significantly different from the control group (**Figures 2A–C**). In the comparison of iohexol to the control group, 1391 DEGs were discovered, comprising 1031 up-regulated DEGs and 360 down-regulated DEGs. While 3077 DEGs were found in the comparison of cisplatin with the control group including 1504 up-regulated DEGs and 1573 down-regulated DEGs. 3336 DEGs were also found in the comparison of vancomycin with the control group including 1941 up-regulated DEGs and 1395 down-regulated DEGs. The results of each group were presented in the form of Volcano plot (**Figures 2D–F**). The top 50 genes with low *P-values* were presented as a heatmap (**Figures 2G–I**).

### 3.2 Functional enrichment of DEGs and protein-protein network construction

**Figure 3:**
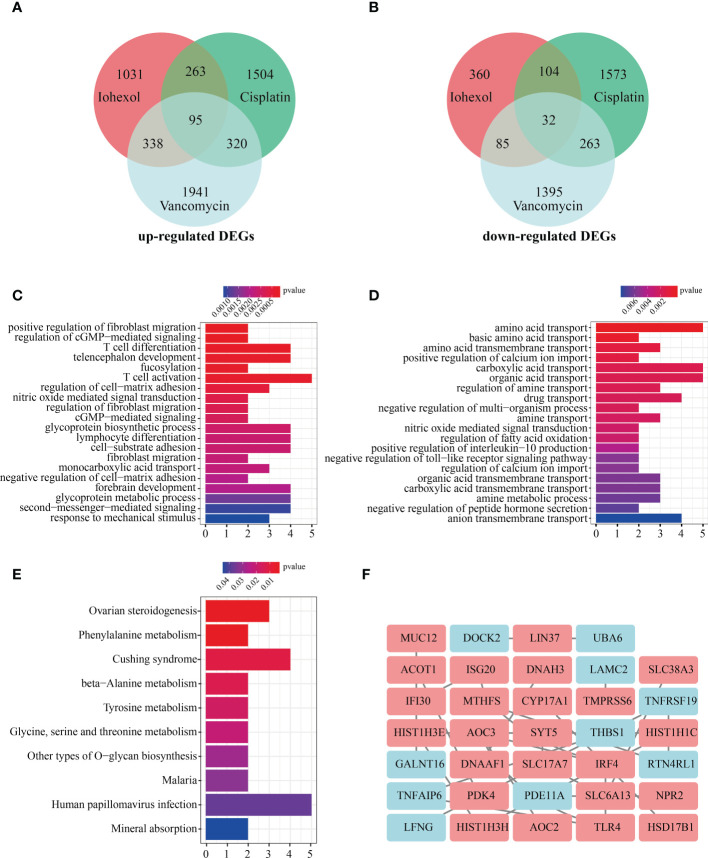
Enrichment analysis of co-DEGs and PPI network construction. **(A)**Vene diagram indicates intersection of up-regulated DEGs in three AKI datasets. **(B)** Intersection of down-regulated DEGs in three AKI datasets. **(C)** The GO enrichment (BP, CC and MF) of up-regulated co-DEGs. **(D)** The GO enrichment (BP, CC and MF) of down-regulated co-DEGs. **(E)** KEGG analysis of co-DEGs. **(F)** PPI network construction of 34 co-DEGs interaction with each other. Upregulated co-DEGs are marked in red while downregulated co-DEGs are marked in blue BP, biological process; CC, cellular component; MF, molecular function; KEGG, Kyoto Encyclopedia of Genes and Genomes; PPI, protein-protein interaction network.

The intersection of up-regulated DEGs or down-regulated DEGs of the three groups of drugs was computed. A total 95 co-up-regulated and 32 down-regulated DEGs were identified and displayed using Venn diagram (**Figures 3A, B**). To figure out the functions of co-DEGs, “clusterprofiler”, was used for GO enrichment (BP, CC, and MF). The co-upregulated genes were mostly enriched in functions like positive regulation of fibroblast migration, regulation of cGMP-mediated signaling, T cell differentiation and T cell activation (**Figure 3C**). However, the co-down-regulated genes were associated with amino acid transport, positive regulation of calcium ion import, carboxylic acid transport and drug transport (**Figure 3D**). KEGG pathway analysis showed that co-DEGs were related to Ovarian steroidogenesis, Phenylalanine metabolism, Cushing syndrome and beta-Alanine metabolism (**Figure 3E**). The co-DEGs were then utilized to build the PPI network for future investigation. The findings reveal that a PPI network with 84 nodes and 24 edges was built with confidence >0.4 (**Figure 3F**).

### 3.3 Identification of hub genes and their expression in various datasets

**Figure 4:**
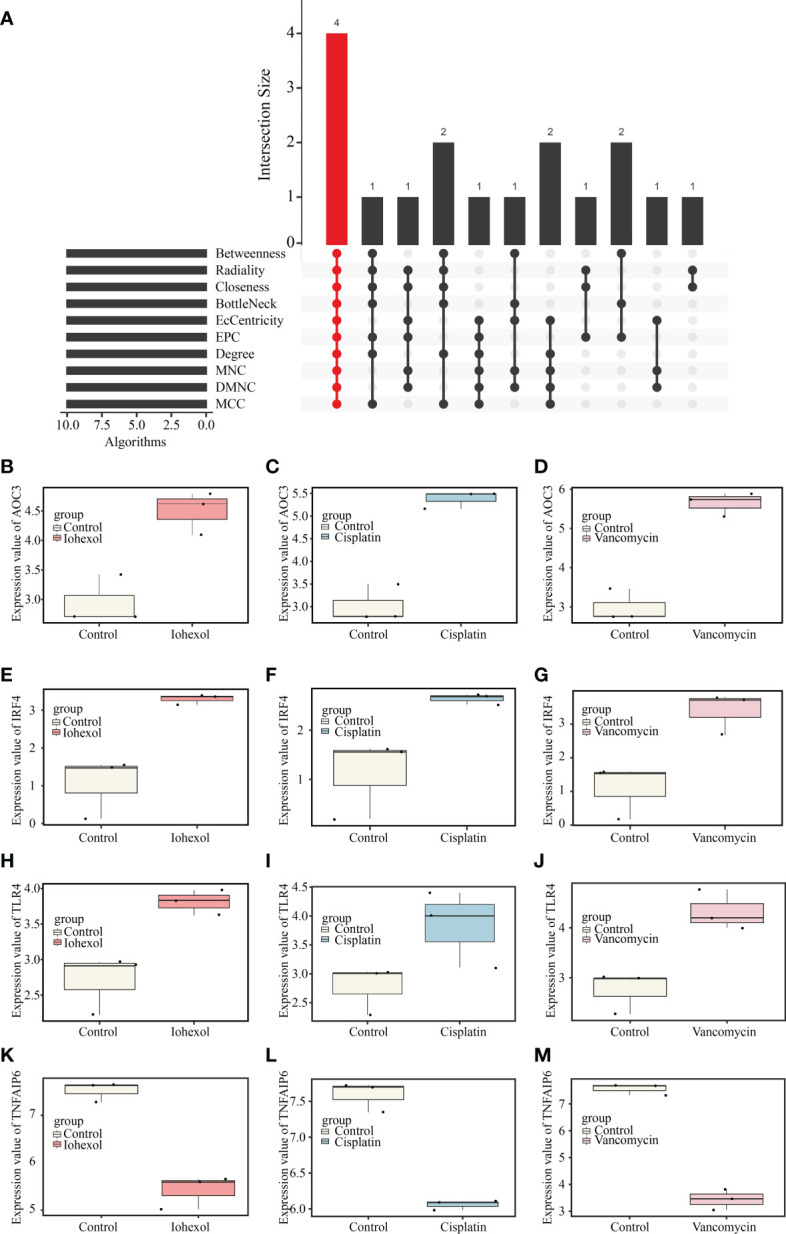
Identification of hub genes and their expression in three AKI datasets. **(A)** Four hub genes (AOC3, IRF4, TLR4 and TNFAIP6) were identified by intersection of co-DEGs from 10 algorithms, namely, MCC, DMNC, MNC, Degree, EPC, BottleNeck, EcCentricity, Closeness, Radiality, and Betweenness. **(B–D)** The expression value of AOC3 in iohexol, cisplatin or vancomycin datasets compared with controls. **(E–G)** The expression value of IRF4 in iohexol, cisplatin or vancomycin datasets compared with controls. **(H–J)** The expression value of TLR4 in iohexol, cisplatin or vancomycin datasets compared with controls. **(K-M)** The expression value of TNFAIP6 in iohexol, cisplatin or vancomycin datasets compared with controls.

Ten different algorithms were used to calculated the most precise common hub genes and intersected them in order to determine intersecting genes. AOC3, IRF4, TLR4, and TNFAIP6 were eventually identified as hub genes (**Figure 4A**). The details of these four genes are presented in **Table 3**. In Compared to control group, the expression level of AOC3, IRF4 and TLR4 were increased after treatment with the three drugs respectively, while the expression of TNFAIP6 was the opposite. The expression level of AOC3, IRF4 or TLR4 in iohexol group, cisplatin group and vancomycin group was visually depicted by boxplots respectively (**Figures 4B–J**). Similarly, the expression of and TNFAIP6 in iohexol group, cisplatin group and vancomycin group were separately shown in boxplots (**Figures 4K–M**).

**Table 3:** Information for the hub genes.

Gene symbol	Full name	Synonyms	Function
AOC3	Amine Oxidase Copper Containing 3	VAP-1	Cell adhesion protein that participates in lymphocyte extravasation and recirculation by mediating the binding of lymphocytes to peripheral lymph node vascular endothelial cells in an L-selectin-independent fashion.
IRF4	Interferon Regulatory Factor 4	LSIRF, MUM1	Transcriptional activator. Binds to the interferon-stimulated response element (ISRE) of the MHC class I promoter. Binds the immunoglobulin lambda light chain enhancer, together with PU.1. Probably plays a role in ISRE-targeted signal transduction mechanisms specific to lymphoid cells.
TLR4	Toll Like Receptor 4	HToll CD284	Cooperates with LY96 and CD14 to mediate the innate immune response to bacterial lipopolysaccharide (LPS); Acts *via* MYD88, TIRAP and TRAF6, leading to NF-kappa-B activation, cytokine secretion and the inflammatory response
TNFAIP6	TNF Alpha Induced Protein 6	TSG-6	Major regulator of extracellular matrix organization during tissue remodeling;

### 3.4 Validation of hub genes *in vitro*

**Figure 5:**
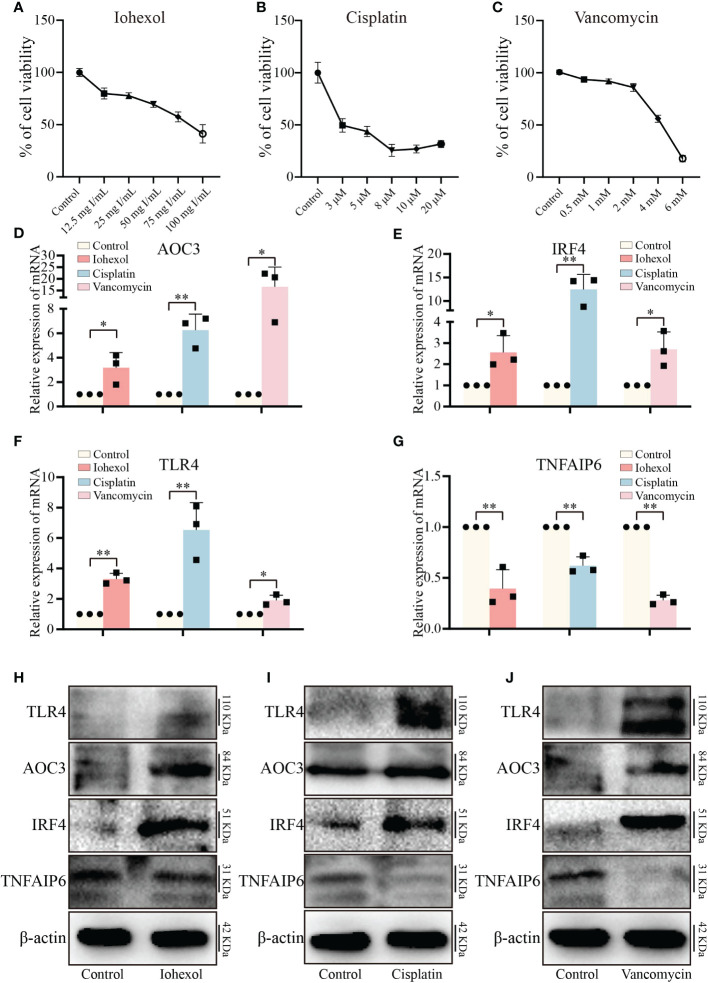
Validation of hub genes *in vitro*. **(A–C)** The variations in relative cell viability of HK-2 cells as the concentration of iohexol, cisplatin or vancomycin dosage increased. **(D)** The mRNA expression level of AOC3 under the treatment of iohexol, cisplatin or vancomycin compared with controls. **(E)** The mRNA expression level of IRF4 under the treatment of iohexol, cisplatin or vancomycin compared with controls. **(F)** The mRNA expression level of TLR4 under the treatment of iohexol, cisplatin or vancomycin compared with controls. **(G)** The mRNA expression level of TNFAIP6 under the treatment of iohexol, cisplatin or vancomycin compared with controls. **(H)** Representative pictures of the AOC3, IRF4, TLR4, and TNFAIP6 protein levels of the iohexol-induced AKI *in vitro*. **(I)** Representative pictures of the AOC3, IRF4, TLR4, and TNFAIP6 protein levels of the cisplatin-induced AKI *in vitro*. **(J)** Representative pictures of the AOC3, IRF4, TLR4, and TNFAIP6 protein levels of the vancomycin-induced AKI *in vitro*. Each result was repeated at least three times. Compared with the control group, *p <0.05; **p <0.01.

To validate the toxicity of these medications to HK2 cells and establish the cell DI-AKI models, cell viability was measured at various doses of iohexol, cisplatin, or vancomycin. The results showed that cell viability reduced as the medications concentration rose (**Figures 5A–C**). Then, the mRNA level of those four hub genes AOC3, IRF4, TLR4 and TNFAIP6 were detected. The real time PCR results showed that the expression of AOC3, IRF4 and TLR4 in iohexol, cisplatin or vancomycin groups were significantly up-regulated compared with the control group (**Figures 5D–F**). While TNFAIP6 was on the opposite (**Figure 5G**). The protein levels of four hub genes were also verified using western blot (WB), and shown to be the same as variations in mRNA expression (**Figures 5H–J**). Since the results obtained by real time PCR and WB were consistent with our sequencing results, indicating that the hub genes may play roles in drug-induced renal cell injury *in vitro*.

### 3.5 Model establishment and injury evaluation of drug-induced AKI mice

**Figure 6:**
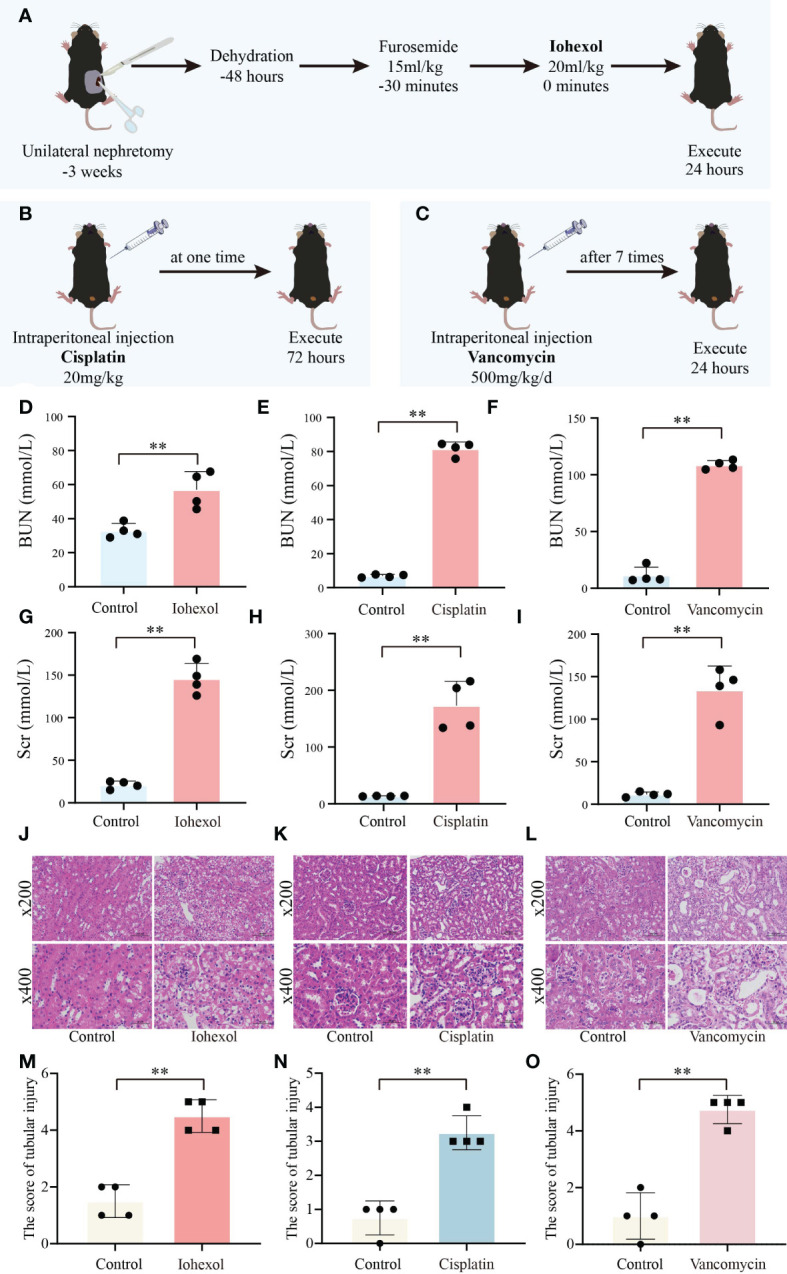
Construction and validation of AKI models. **(A)** Flow chart of iohexol-induced AKI model construction. **(B)** Flow chart of cisplatin-induced AKI model construction. **(C)** Flow chart of vancomycin-induced AKI model construction. **(D–F)** Compared to controls, BUN levels in AKI mice induced by iohexol, cisplatin, or vancomycin. **(G–I)** Compared to controls, Scr levels in AKI mice induced by iohexol, cisplatin, or vancomycin. BUN, blood urea nitrogen; Scr, serum creatinine. **(J–O)** Representative picture of HE staining and the kidney injury scores of controls, iohexol, cisplatin or vancomycin induced AKI mice. Each result was repeated four times. Compared with the control group, **p <0.01.

To further verify the role of hub genes *in vivo*, we constructed three AKI mice model including iohexol-induce AKI mice, cisplatin-induced AKI mice and vancomycin-induced AKI mice in accordance with the prior technical basis of our laboratory (**Figures 6A–C**). Then we confirmed that these models were successfully established through detected BUN and Scr of serum from AKI mice. Both indicators are gold indexes for evaluating renal injury (**Figures 6D–I**). HE staining and its tubule injury score also demonstrated the damage of drugs to the kidney (**Figures 6J–O**). The results demonstrated that the AKI models were successfully constructed.

### 3.6 Validation of hub genes *in vivo*

**Figure 7:**
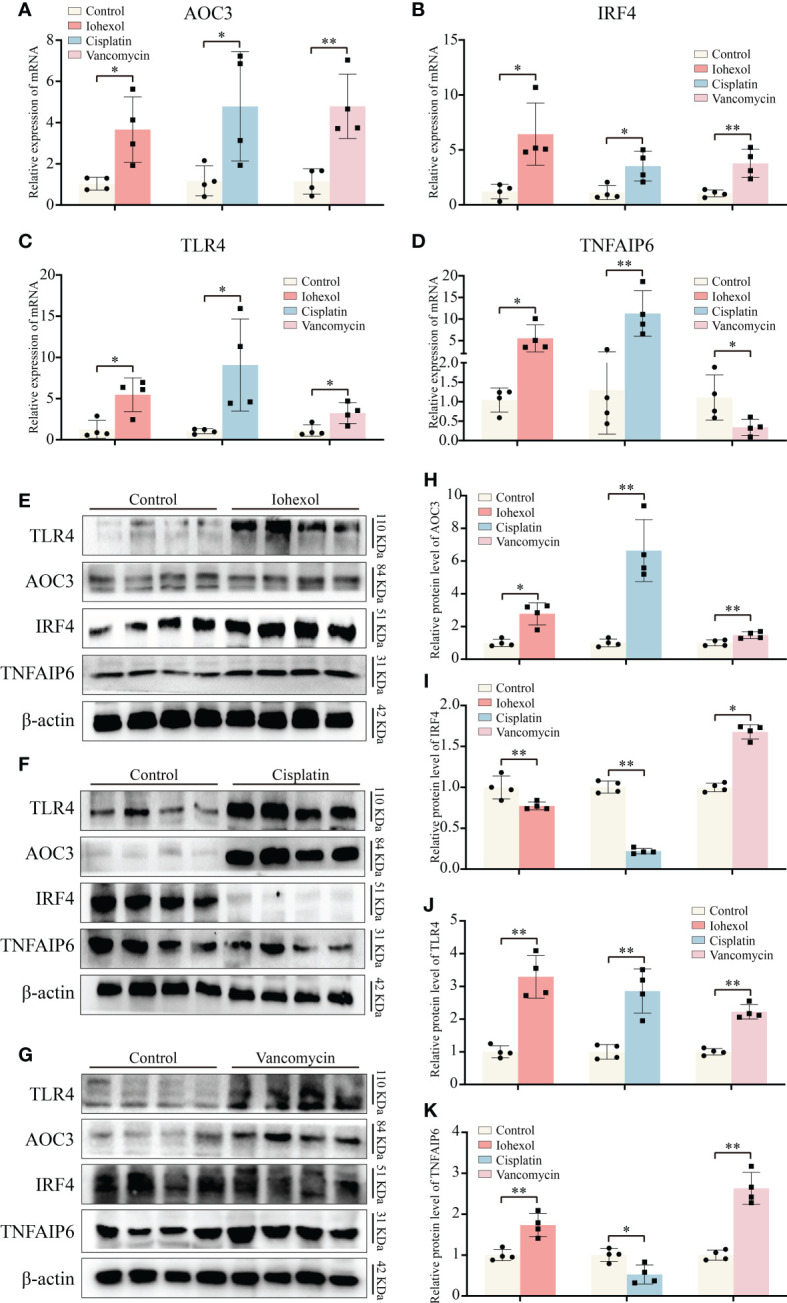
Validation of hub genes *in vivo*. **(A–D)** The mRNA expression level of AOC3, IRF4, TLR4 and TNFAIP6 in renal tissue of iohexol, cisplatin or vancomycin induced AKI mice compared with controls. **(E–K)** The protein expression level of AOC3, IRF4, TLR4 and TNFAIP6 in renal tissue of iohexol, cisplatin or vancomycin induced AKI mice compared with controls. *p <0.05; **p <0.01.

By extracting mRNA from kidney tissue of different groups of mice, we then verified the *in vivo* expression of the four hub genes. The results of mRNA showed that, similar to the *in vitro* validation results, AOC3, IRF4 and TLR4 were highly expressed in iohexol, cisplatin or vancomycin groups compared to control group (**Figures 7A–C**). Interestingly, TNFAIP6 was also highly expressed in the iohexol or cisplatin group. However, in the vancomycin group, the expression of TNFAIP6 was down-regulated, consistent with the *in vitro* results (**Figure 7D**).

Next, we extracted proteins from the kidneys of DI-AKI mice to further verify the expression levels of the four hub genes *in vivo* (**Figures 7E–G**). Among them, TLR4 and AOC3 were significantly increased in iohexol, cisplatin and vancomycin mouse models, which were the same as mRNA levels (**Figures 7H, I**). IRF4 and TNFAIP6 protein alterations, however, were distinct from mRNA expression changes. IRF4 protein levels were decreased by both cisplatin and vancomycin, while TNFAIP6 rose in vancomycin-treated mice while it dropped in cisplatin-treated ones (**Figures 7J, K**). The mechanisms that lead to this need to be further explored. In general, AOC3, and TLR4 showed the same *in vivo* and *in vitro* performance after different drug treatments, which further reflected the role of these hub genes in Drug-induced AKI.

### 3.7 Verification of hub genes in mouse serum and urine

**Figure 8:**
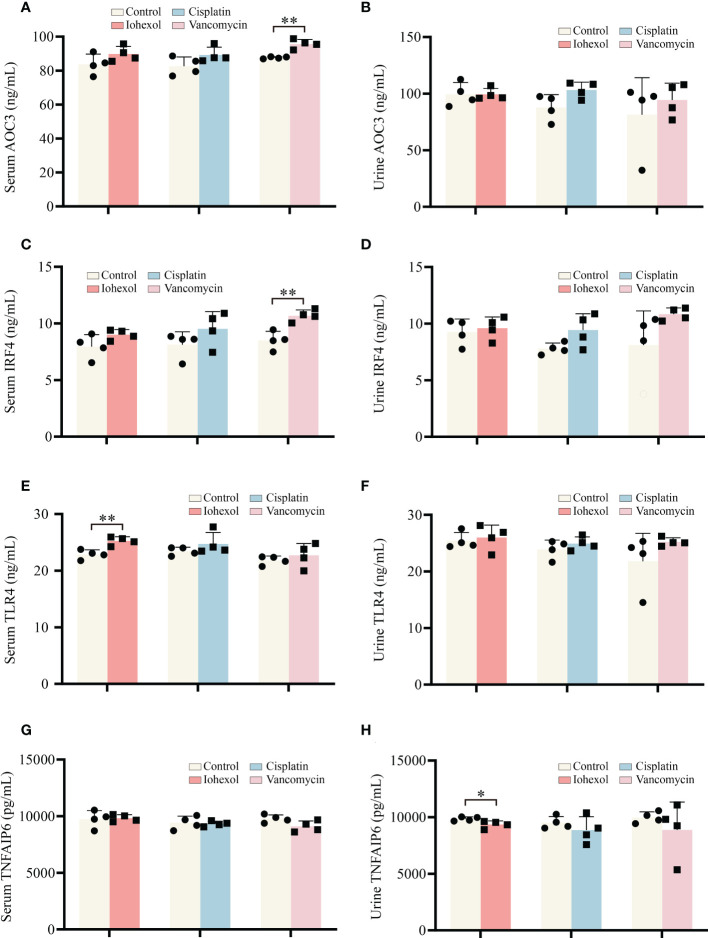
Validation of the hub genes in serum and urine of DI-AKI mice. **(A, B)** AOC3 protein levels in serum and urine of DI-AKI mice. **(C, D)** IRF4 protein levels in serum and urine of DI-AKI mice. **(E, F)** TLR4 protein levels in serum and urine of DI-AKI mice. **(G, H)** TNFAIP6 protein levels in serum and urine of DI-AKI mice. Compared with the control group, *p <0.05; **p <0.01.

Further research to determine whether significant hub genes have potential as biomarkers is helpful for the early detection of DI-AKI. ELISA kits were used to assess the protein concentrations in mouse serum and urine. TLR4, IRF4, and AOC3 exhibited an increasing trend in serum and urine of DI-AKI mice, while TNFAIP6 showed a lowering trend, according to the **Figure 8**. In mice with iohexol-induced AKI, the serum TLR4 level was markedly elevated. Serum from vancomycin-induced AKI mice contained considerably higher levels of AOC3 and IRF4. TNFAIP was significantly reduced in the urine of mice with AKI brought on by iohexol. However, other findings were not statistically significant. These findings imply that TLR4, IRF4, AOC3, and TNFAIP6 may be useful as DI-AKI biomarkers, and more research is required to determine their effectiveness.

### 3.8 Verification of hub genes by GEO datasets

**Figure 9:**
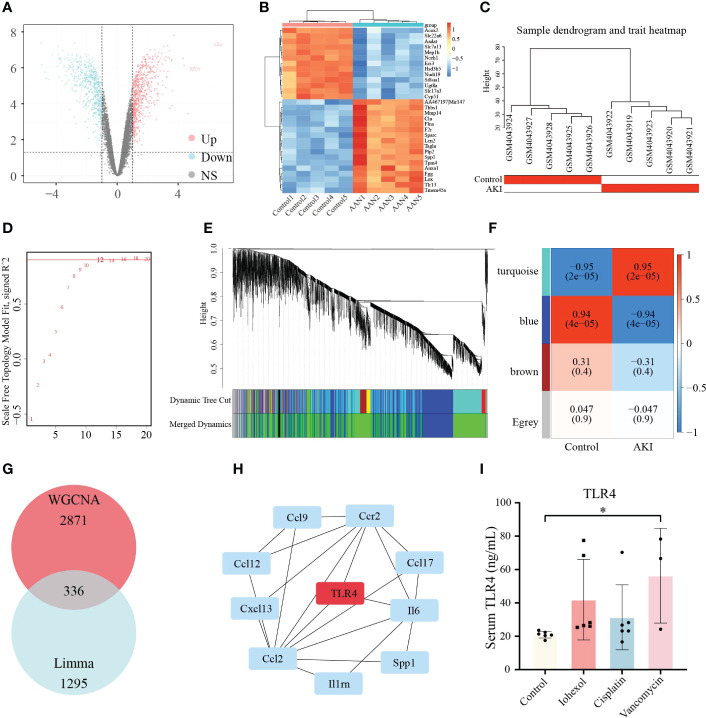
Validation of the hub genes in AAN datasets. **(A)** Volcano plots of DEGs identified from AAN datasets. Red dots represent up-regulated DEGs whereas blue dots represent down-regulated DEGs. **(B)** Heatmap of top 30 DEGs rated by *P-Value* identified in AAN datasets. **(C)** Sample dendrogram and trait heatmap show the degree of outliers and grouping information of samples. **(D)** The “soft” threshold based on the scale independence and average connectivity. **(E)** Gene co-expression modules represented by different colors under the hierarchical clustering tree. **(F)** Heatmap of the association between modules and AKI. The turquoise module was shown to be correlated significantly with AKI. Numbers at the top and bottom brackets represent the correlation coefficient and *P-value*, respectively. **(G)**The Venn diagram shows the intersection of genes in turquoise module and DEGs. **(H)** The line diagram indicates first ten hub genes results obtained by EcCentricity algorithm in Cytohubba plugin. **(I)** TLR4 protein levels in patients with iohexol-, cisplatin- and vancomycin- induced AKI compared with healthy controls. Compared with the control group, *p <0.05.

Due to the complexity of the AKI models, we attempted to employ additional AKI models to demonstrate the relevance between hub genes we screened and DI-AKI. Limma package was used to find DEGs of AAN mice. The results were shown in form of Volcano plot and Heatmap (**Figures 9A, B**). Meanwhile, WGCNA was also used to explore the correlation between genes. A sample cluster map was created to figure out there are any outlier samples (**Figure 9C**). Then, we chose β = 12 (scale-free R^2^ = 0.9) as the “soft” threshold based on the scale independence and average connectivity (**Figure 9D**). By calculating the adjacency matrix and drawing the hierarchical clustering tree, the modules are obtained, and then the modules with similarity greater than 0.75 are merged to reduce the complexity of the network (**Figure 9E**). The correlation between Control and AKI was shown in **Figure 9F** with the turquoise module (2871 genes) demonstrated the highest correlation with AKI (correlation coefficient = 0.95, p = 2.0 * 10^-5^) and was regarded as the pivotal module for subsequent analysis. Furthermore, genes significantly associated with AKI obtained by WGCNA and DEGs identified by LIMMA package were intersected, and 336 genes were obtained (**Figure 9G**). These 336 genes were then input into the String database to obtain a PPI network with 312 nodes and 459 edges (high confidence = 0.70) (**Table S1**). Next, Cytohubba was used to search for hub genes, and TLR4 was found in at least three algorithms (**Figures S1**, **2**). Immunohistochemical staining on the kidneys of DI-AKI mice was done to further confirm the change level of TLR4, and it was once again confirmed that the level of TLR4 was dramatically elevated in DI-AKI animals (**Figure S3**). The first ten hub genes results obtained by EcCentricity algorithm was shown in **Figure 9H**. Surprisingly, when we searched for DEGs of AAN data, we found that AOC3, IRF4 and TNFAIP6 were also slightly up-regulated compared with the control group (**Table S2**). Also, TLR4, AOC3, IRF4, and TNFAIP6 were not differentially expressed in non-drug-induced AKI models including IRI (**Table S3**) and UUO (**Table S4**) models, which was unexpected and additional evidence of the specificity of these hub genes in drug-induced kidney damage.

### 3.9 Verification of TLR4 in serum of in clinical samples

Based on sequencing and validation of cell, mouse, and aristolochic acid databases, we found that TLR4 could be the most valuable marker among them. Therefore, TLR4 levels in clinical practice we detected by ELISA kit in collected patient samples. Twenty-one human plasma samples were obtained, together with the related basic data (age, gender, etc.), and the basic data was examined by single factor analysis. It was discovered that there was no discernible difference between DI-AKI and Control groups in the fundamental situation (**Table 4**). In **Table S5**, further specific patient features are displayed. The results showed that TLR4 levels were increased in serum samples from iohexol-, cisplatin-, and vancomycin-induced AKI patients (**Figure 9I**). Due to the small sample size and significant intra-group variation, only patients with vancomycin-induced AKI showed a statistically significant difference as a result. More samples and studies are needed to further determine whether TLR4 can be used as a biomarker for DI-AKI.

**Table 4:** Basic information and single factor analysis between DI-AKI group and Non-DI-AKI group.

Characteristics	Control (N=6, Mean ± SD)	DI-AKI (N=15, Mean ± SD)	Rval.x	Pval.x
Age	55.00 ± 11.40	55.60 ± 11.46	0.00	0.92
Meal sex (No, %)	4 (66.67)	8 (53.33)	0.56	0.58
Weight	63.33 ± 16.39	60.41 ± 9.37	0.04	0.39
Hight	161 ± 8.97	160.83 ± 7.25	-0.03	0.68
BMI	24.90 ± 3.38	22.70 ± 3.68	0.18	0.20
Scr1	72.17 ± 18.09	111.73 ± 183.30	0.00	0.64

## 4 Discussion

Drug use is one of the prevalent causes of acute kidney injury, accounting for approximately 14%-26% of AKI in adults (2). Since no specific effective treatment for DI-AKI, early identification and diagnosis of renal toxicity are essential for prevention (6). However, the application of existing common markers of kidney injury in DI-AKI is insufficient, and further exploration of new biomarkers of DI-AKI is required to increase early diagnosis and detection of DI-AKI and better prognosis (7, 13). Therefore, DI-AKI cell models were constructed using iohexol, cisplatin and vancomycin respectively, and transcriptomic sequencing was performed on them. Four key hub genes (TLR4, AOC3, IRF4, and TNFAIP6) were predicted by bioinformatics methods, and the importance of hub genes was verified in cells, mice, human specimens, and public databases. These results may provide new strategies for diagnosing and treating patients with DI-AKI earlier and more accurately.

Currently, several studies have reported the potential mechanism of AKI caused by cisplatin, iohexol and vancomycin. Cisplatin hydrolysis generates positively charged metabolites that have the potential to seriously harm DNA, particularly mitochondrial DNA. In addition to DNA damage, cisplatin causes dysfunction of cytoplasmic organelles, particularly in the endoplasmic reticulum and mitochondria, activates apoptotic pathways, and causes cellular damage through oxidative stress and inflammation, leading to renal insufficiency (16, 26). It is now considered that CM cause mitochondrial dysfunction, apoptosis and necrosis in tubular epithelial cells *via* direct nephrotoxicity while its indirect impact alters renal hemodynamics and triggers medulla hypoxia, resulting in an increase of ROS which leads oxidative stress and inflammation. Both of these factors work together to impair renal function (17, 27, 28). The exact pathophysiology of vancomycin-induced AKI is not fully understood. According to the current consensus, vancomycin accumulates inside cells, causing oxidative stress, complement activation, inflammatory damage, mitochondrial malfunction, and death in the proximal renal tubules (20, 29). Our results identified four key related candidate genes (AOC3, IRF4, TLR4 and TNFAIP6) in iohexol-induced, cisplatin-induced and vancomycin-induced AKI models, and validated them *in vitro* and *in vivo*. It’s interesting to note that the four important genes we sequenced and examined were all involved in inflammation and immunity. We further discovered that among these, TLR4 alteration were most compatible with the sequencing of aristolochic acid AKI mouse.

TLR4, an evolutionarily conserved innate immune receptor, is involved in the detection and transmission of danger signals, such as pathogen-related molecular patterns, damage-related molecular patterns and xenobiology-related molecular patterns (30). Inflammation is an early response to tissue damage and involves a large number of innate immune cells with TLR4 being the primary factor promoting inflammation (31). It is reported that TLR4 recognizes extracellular HMGB1 and activates downstream signaling *via* MYD88 and TIR-domain-containing adapter-inducing interferon-β, further promoting nuclear translocation of NF-κB and increasing levels of inflammatory cytokines and chemokines (32). TLR4 was shown abundantly expressed in all renal cell types, including renal tubular epithelial cells and podocytes (33). By promoting inflammation, programmed cell death, and endoplasmic reticulum stress, TLR4 has been linked to several acute and chronic renal disorders (34). Studies on contrast-induced AKI have found that contrast media increased the expression of TLR4 and up-regulate TLR4-related downstream inflammatory pathways (35, 36). Ginsenoside Rb1/atorvastatin both attenuate renal injury by inhibiting the activation of TLR4)/NF-κB signaling pathway triggered by contrast media (37, 38). In cisplatin induced AKI, Zhang et al. demonstrated that renal parenchymal TLR4, but not bone marrow TLR4, mediates the nephrotoxic effects of cisplatin. Activation of TLR4 on renal parenchymal cells may activate the p38 MAPK pathway, leading to increased production of inflammatory cytokines such as TNF-α and subsequent renal injury (39). Salvianolic acid C (40), Cordyceps cicadae Mycelia (41) reduce the expression level of TLR-4 and enhance several antioxidant enzymes (superoxide dismutase and glutathione peroxidase) to alleviate cisplatin induced AKI. Consistent with the above results, our results showed that TLR4 mRNA levels were significantly elevated in cells and mouse models of DI-AKI. Also, it was discovered that TLR4 was considerably enhanced only in AAN (a DI-AKI model), but not in IRI or UUO models, by evaluating the sequencing data of these three mouse strains in the GEO database. This finding raises the idea that TLR4 has a DI-AKI-specific biomarker. Most noteworthy, despite only vancomycin-induced AKI showing a statistically significant difference, TLR4 levels were considerably higher in patients with DI-AKI. We thus hypothesize that TLR4 has specific biomarker potential in diverse DI-AKI, which is deserving of future investigation based on our findings and the bulk of current research.

IRF4 is a multifunctional transcription factor closely related to inflammation and fibrous diseases (42). Study has shown that folic acid treatment resulted in severe acute tubule injury in mice, and a sharp increase in IRF4 levels was observed in FA-damaged kidneys. Similarly, in our results, IRF4 was significantly elevated in DI-AKI. IRF4^-/-^ reduces folate AKI induced inflammation and renal fibrosis (43). Moreover, IRF4 is a regulator of adaptive immunity, which is necessary for T cell and B cell maturation to regulate T cell function (44). It also binds to MyD88 and inhibits TLRs signaling (42). By interacting with MyD88, IRF4 can prevent TLR2 and TLR4 signals from interacting with downstream signal components, therefore controlling innate immunity (45). These findings imply that IRF4 has some promise as a biomarker of DI-AKI and that more research into the IRF4/TLR4 pathway in DI-AKI is warranted.

AOC3 is a unique molecule that serves both as an adhesion molecule and as an exoenzyme to catalyze oxidative deamination of primary amines and to produce hydrogen peroxide in the extracellular space, and VPA-1 has been implicated in various inflammatory diseases (46). VAP-1 plays a key role in the pathophysiology of renal ischemia/reperfusion injury by enhancing neutrophil infiltration to produce a local hydrogen peroxide gradient (46). Furthermore, serum VAP-1 has been found to be higher in patients with diabetes and chronic kidney disease (CKD) and to predict cardiovascular mortality in patients with diabetes (47). VAP-1 inhibitor ASP8232 reduces albuminuria in patients with type 2 diabetes and chronic kidney disease in Phase II clinical trials (48, 49). Our results also demonstrate that AOC3 was highly expressed in DI-AKI. Therefore, we believe that ACO3 may play a role in DI-AKI by promoting immune cell infiltration and inflammation, and serve as a potential marker.

TNFAIP6 is an inflammation-related secreted protein which is upregulated in the presence of inflammation and thought to have important and diverse tissue protective and anti-inflammatory properties (50, 51). It is reported that TNFAIP6 can reduce AKI after paraquat poisoning by inhibiting the inflammatory response (52). Similarly, we found that the mRNA expression of TNFAIP6 in the cell models were significantly down-regulated by the medications. Surprisingly, our verification in the DI-AKI mouse model was not the same. Among them, the TNFAIP6 level in kidney tissues of AKI mice induced by vancomycin was significantly decreased. On the contrary, iohexol and cisplatin-induced AKI mice had higher levels of TNFAIP6 in their renal tissues. This could be as a result of the various model establishment cycles, with vancomycin requiring a 7-day period of continuous treatment to build the model. The damage caused by iohexol and cisplatin over the course of 72 hours may have caused the elevation of TNFAIP6. Similarly, Cox et al. showed that the expression of TNFAIP6 protein in glomerular and tubule cells was significantly up-regulated in IgA nephropathy (IGAN) patients compared with non-IGAN patients and controls (53). Therefore, the role of TNFAIP6 in DI-AKI needs to be further determined.

Our study has several limitations. Despite the fact that we merged data sets from four separate DI-AKI models, only the contrast media, anticancer drugs, antibacterial agents, and traditional Chinese medicine components are represented. Other medications also have the potential to harm the kidneys, so more research on AKI brought on by other medications is required. Also, the sample size for sequencing and validation is modest, and additional samples, particularly human specimens, are required to further validate the findings. Furthermore, we did not evaluate the efficiency of these hub genes in DI-AKI by comparing them to conventional biomarkers, hence, additional sample collection and analysis are required. Finally, the alterations of these important hub genes in the non-DI-AKI model were merely confirmed using the data, however, additional experimental confirmation is still required.

In this study, we identified four essential DI-AKI Hub genes (TLR4, AOC3, IRF4, and TNFAIP6) using mRNA sequencing and extensive analysis of the three conventional DI-AKI models. Unexpectedly, all of these genes have a relationship with immunity and inflammation. These four core Hub genes might be used as potential biomarkers and might present a novel approach to the clinical diagnosis and management of AKI. According to validation at cellular level, mouse level, human level, and databases (AAN, IRI and UUO), TLR4 may have the most potential among these key Hub genes.

## Data availability statement

The data presented in the study are deposited in the GEO repository, accession number GSE227970. https://www.ncbi.nlm.nih.gov/geo/query/acc.cgi?acc=GSE227970.

## Ethics statement

The studies involving human participants were reviewed and approved by the Ethics Committee of the Third Xiangya Hospital of Central South University. The patients/participants provided their written informed consent to participate in this study. The animal study was reviewed and approved by Medical Research Ethics Committee of Central South University. Written informed consent was obtained from the individual(s) for the publication of any potentially identifiable images or data included in this article.

## Author contributions

Funding acquisition: X-CZ and L-YZ. Investigation: Y-XD, KL and X-RG. Software: Y-XD. Study materials: KL, Q-XQ, Z-YT, G-LZ and Y-FW. Human specimen collection: R-MQ, D-KL and W-JY. Supervision: X-CZ. Validation: Y-XD and KL. Writing original draft: Y-XD and KL. All authors contributed to the article and approved the submitted version.

## Supplementary material

Supplementary Figure 1 The line diagram indicates first ten Hub genes results obtained by BottleNeck algorithm in Cytohubba plugin.

Supplementary Figure 2 The line diagram indicates first ten Hub genes results obtained by EPC algorithm in Cytohubba plugin.

Supplementary Figure 3 Representative image of TLR4 immunohistochemistry in AKI mouse kidneys induced by iohexol, cisplatin and vancomycin.

## References

[1] Pierson-Marchandise M Gras V Moragny J Micallef J Gaboriau L Picard S. The drugs that mostly frequently induce acute kidney injury: A case - noncase study of a pharmacovigilance database. Br J Clin Pharmacol (2017) 83(6):1341–9. doi: 10.1111/bcp.13216 PMC5427222 28002877

[2] Hoste EA Bagshaw SM Bellomo R Cely CM Colman R Cruz DN. Epidemiology of acute kidney injury in critically ill patients: the multinational AKI-EPI study. Intensive Care Med (2015) 41(8):1411–23. doi: 10.1007/s00134-015-3934-7 26162677

[3] Kwiatkowska E Domański L Dziedziejko V Kajdy A Stefańska K Kwiatkowski S. The mechanism of drug nephrotoxicity and the methods for preventing kidney damage. Int J Mol Sci (2021) 22(11):6109. doi: 10.3390/ijms22116109 34204029 PMC8201165

[4] Perazella MA Rosner MH. Drug-induced acute kidney injury. Clin J Am Soc Nephrol (2022) 17(8):1220–33. doi: 10.2215/CJN.11290821 PMC9435983 35273009

[5] Yang L Xing G Wang L Wu Y Li S Xu G. Acute kidney injury in China: A cross-sectional survey. Lancet (2015) 386(10002):1465–71. doi: 10.1016/S0140-6736(15)00344-X 26466051

[6] Khan S Loi V Rosner MH. Drug-induced kidney injury in the elderly. Drugs Aging (2017) 34(10):729–41. doi: 10.1007/s40266-017-0484-4 28815461

[7] Blank M Thompson A Hausner E Rouse R. Biomarkers of drug-induced acute kidney injury: a regulatory perspective. Expert Opin Drug Metab Toxicol (2018) 14(9):929–36. doi: 10.1080/17425255.2018.1511701 30099912

[8] Devarajan P. Biomarkers for the early detection of acute kidney injury. Curr Opin Pediatr (2011) 23(2):194–200. doi: 10.1097/MOP.0b013e328343f4dd 21252674 PMC3257513

[9] Waikar SS Betensky RA Bonventre JV. Creatinine as the gold standard for kidney injury biomarker studies? Nephrol Dial Transplant (2009) 24(11):3263–5. doi: 10.1093/ndt/gfp428 19736243

[10] Kim SM Lee HS Kim MJ Park HD Lee SY. Diagnostic value of multiple serum biomarkers for vancomycin-induced kidney injury. J Clin Med (2021) 10(21):5005. doi: 10.3390/jcm10215005 34768522 PMC8584616

[11] Sampaio de Souza Garms D Cardoso Eid KZ Burdmann EA Marcal LJ Antonangelo L Dos Santos A. The role of urinary biomarkers as diagnostic and prognostic predictors of acute kidney injury associated with vancomycin. Front Pharmacol (2021) 12:705636. doi: 10.3389/fphar.2021.705636 34630082 PMC8495315

[12] Valette X du Cheyron D. Accuracy of plasma neutrophil gelatinase-associated lipocalin in the early diagnosis of contrast-induced acute kidney injury in critical illness: reply to quartin et al. Intensive Care Med (2013) 39(12):2239. doi: 10.1007/s00134-013-2826-y 24081434

[13] Wu H Huang J. Drug-induced nephrotoxicity: Pathogenic mechanisms, biomarkers and prevention strategies. Curr Drug Metab (2018) 19(7):559–67. doi: 10.2174/1389200218666171108154419 29119923

[14] Perazella MA. Drug-induced acute kidney injury: diverse mechanisms of tubular injury. Curr Opin Crit Care (2019) 25(6):550–7. doi: 10.1097/MCC.0000000000000653 31483318

[15] Volarevic V Djokovic B Jankovic MG Harrell CR Fellabaum C Djonov V. Molecular mechanisms of cisplatin-induced nephrotoxicity: a balance on the knife edge between renoprotection and tumor toxicity. J BioMed Sci (2019) 26(1):25. doi: 10.1186/s12929-019-0518-9 30866950 PMC6417243

[16] Fang CY Lou DY Zhou LQ Wang JC Yang B He QJ. Natural products: potential treatments for cisplatin-induced nephrotoxicity. Acta Pharmacol Sin (2021) 42(12):1951–69. doi: 10.1038/s41401-021-00620-9 PMC8633358 33750909

[17] Mehran R Dangas GD Weisbord SD. Contrast-associated acute kidney injury. N Engl J Med (2019) 380(22):2146–55. doi: 10.1056/NEJMra1805256 31141635

[18] Kusirisin P Chattipakorn SC Chattipakorn N. Contrast-induced nephropathy and oxidative stress: mechanistic insights for better interventional approaches. J Transl Med (2020) 18(1):400. doi: 10.1186/s12967-020-02574-8 33081797 PMC7576747

[19] McCullough PA Choi JP Feghali GA Schussler JM Stoler RM Vallabahn RC. Contrast-induced acute kidney injury. J Am Coll Cardiol (2016) 68(13):1465–73. doi: 10.1016/j.jacc.2016.05.099 27659469

[20] Kan WC Chen YC Wu VC Shiao CC. Vancomycin-associated acute kidney injury: A narrative review from pathophysiology to clinical application. Int J Mol Sci (2022) 23(4):2052. doi: 10.3390/ijms23042052 35216167 PMC8877514

[21] Sborchia M De Prez EG Antoine MH Bienfait L Indra R Valbuena G. The impact of p53 on aristolochic acid I-induced nephrotoxicity and DNA damage in vivo and *in vitro* . Arch Toxicol (2019) 93(11):3345–66. doi: 10.1007/s00204-019-02578-4 PMC6823306 31602497

[22] Ritchie ME Phipson B Wu D Hu Y Law CW Shi W. Limma powers differential expression analyses for RNA-sequencing and microarray studies. Nucleic Acids Res (2015) 43(7):e47. doi: 10.1093/nar/gkv007 25605792 PMC4402510

[23] Szklarczyk D Gable AL Lyon D Junge A Wyder S Huerta-Cepas J. STRING v11: protein-protein association networks with increased coverage, supporting functional discovery in genome-wide experimental datasets. Nucleic Acids Res (2019) 47(D1):D607–d613. doi: 10.1093/nar/gky1131 30476243 PMC6323986

[24] Smoot ME Ono K Ruscheinski J Wang PL Ideker T. Cytoscape 2.8: new features for data integration and network visualization. Bioinformatics (Oxford, England) (2011) 27(3):431–2. doi: 10.1093/bioinformatics/btq675 PMC3031041 21149340

[25] Lex A Gehlenborg N Strobelt H Vuillemot R Pfister H. UpSet: Visualization of intersecting sets. IEEE Trans Vis Comput Graph (2014) 20(12):1983–92. doi: 10.1109/TVCG.2014.2346248 PMC4720993 26356912

[26] Ozkok A Edelstein CL. Pathophysiology of cisplatin-induced acute kidney injury. BioMed Res Int (2014) 2014:967826. doi: 10.1155/2014/967826 25165721 PMC4140112

[27] Lin Q Li S Jiang N Jin H Shao X Zhu X. Inhibiting NLRP3 inflammasome attenuates apoptosis in contrast-induced acute kidney injury through the upregulation of HIF1A and BNIP3-mediated mitophagy. Autophagy (2021) 17(10):2975–90. doi: 10.1080/15548627.2020.1848971 PMC8525960 33345685

[28] Lin Q Li S Jiang N Shao X Zhang M Jin H. PINK1-parkin pathway of mitophagy protects against contrast-induced acute kidney injury *via* decreasing mitochondrial ROS and NLRP3 inflammasome activation. Redox Biol (2019) 26:101254. doi: 10.1016/j.redox.2019.101254 31229841 PMC6597739

[29] Oktem F Arslan MK Ozguner F Candir O Yilmaz HR Ciris M. In vivo evidences suggesting the role of oxidative stress in pathogenesis of vancomycin-induced nephrotoxicity: protection by erdosteine. Toxicology (2005) 215(3):227–33. doi: 10.1016/j.tox.2005.07.009 16112787

[30] Lin C Wang H Zhang M Mustafa S Wang Y Li H. TLR4 biased small molecule modulators. Pharmacol Ther (2021) 228:107918. doi: 10.1016/j.pharmthera.2021.107918 34171331

[31] Majumder S Pushpakumar S Juin SK Jala VR Sen U. Toll-like receptor 4 mutation protects the kidney from ang-II-induced hypertensive injury. Pharmacol Res (2022) 175:106030. doi: 10.1016/j.phrs.2021.106030 34896544 PMC8755630

[32] Rusai K Sollinger D Baumann M Wagner B Strobl M Schmaderer C. Toll-like receptors 2 and 4 in renal ischemia/reperfusion injury. Pediatr Nephrol (2010) 25(5):853–60. doi: 10.1007/s00467-009-1422-4 20130923

[33] Kot K Kosik-Bogacka D Wojtkowiak-Giera A Kolasa-Wolosiuk A Lanocha-Arendarczyk N. The expression of TLR2 and TLR4 in the kidneys and heart of mice infected with acanthamoeba spp. Parasit Vectors (2020) 13(1):480. doi: 10.1186/s13071-020-04351-4 32958053 PMC7507663

[34] Shelke V Kale A Anders HJ Gaikwad AB. Epigenetic regulation of toll-like receptors 2 and 4 in kidney disease. J Mol Med (Berl) (2022) 100(7):1017–26. doi: 10.1007/s00109-022-02218-y 35704060

[35] Tan X Zheng X Huang Z Lin J Xie C Lin Y. Involvement of S100A8/A9-TLR4-NLRP3 inflammasome pathway in contrast-induced acute kidney injury. Cell Physiol Biochem (2017) 43(1):209–22. doi: 10.1159/000480340 28854431

[36] Wang X Zhou J Yang J Wang S Yang L. Role of TLR4/MyD88/NF-kappaB signaling in the contrast-induced injury of renal tubular epithelial cells. Exp Ther Med (2020) 20(5):115. doi: 10.3892/etm.2020.9243 33005241 PMC7523275

[37] Zhou S Lu S Guo S Zhao L Han Z Li Z. Protective effect of ginsenoside Rb1 nanoparticles against contrast-induced nephropathy by inhibiting high mobility group box 1 Gene/Toll-like receptor 4/NF-kappaB signaling pathway. J BioMed Nanotechnol (2021) 17(10):2085–98. doi: 10.1166/jbn.2021.3163 34706808

[38] Yue R Zuo C Zeng J Su B Tao Y Huang S. Atorvastatin attenuates experimental contrast-induced acute kidney injury: a role for TLR4/MyD88 signaling pathway. Ren Fail (2017) 39(1):643–51. doi: 10.1080/0886022X.2017.1361838 PMC6447912 28805489

[39] Zhang B Ramesh G Uematsu S Akira S Reeves WB. TLR4 signaling mediates inflammation and tissue injury in nephrotoxicity. J Am Soc Nephrol (2008) 19(5):923–32. doi: 10.1681/ASN.2007090982 PMC2386719 18256356

[40] Chien LH Wu CT Deng JS Jiang WP Huang WC Huang GJ. Salvianolic acid c protects against cisplatin-induced acute kidney injury through attenuation of inflammation, oxidative stress and apoptotic effects and activation of the CaMKK-AMPK-Sirt1-Associated signaling pathway in mouse models. Antioxidants (Basel) (2021) 10(10):1620. doi: 10.3390/antiox10101620 34679755 PMC8533075

[41] Deng JS Jiang WP Chen CC Lee LY Li PY Huang WC. Cordyceps cicadae mycelia ameliorate cisplatin-induced acute kidney injury by suppressing the TLR4/NF-kappaB/MAPK and activating the HO-1/Nrf2 and sirt-1/AMPK pathways in mice. Oxid Med Cell Longev (2020) 2020:7912763. doi: 10.1155/2020/7912763 32089779 PMC7026739

[42] Negishi H Ohba Y Yanai H Takaoka A Honma K Yui K. Negative regulation of toll-like-receptor signaling by IRF-4. Proc Natl Acad Sci U.S.A. (2005) 102(44):15989–94. doi: 10.1073/pnas.0508327102 PMC1257749 16236719

[43] Chen M Wen X Gao Y Liu B Zhong C Nie J. IRF-4 deficiency reduces inflammation and kidney fibrosis after folic acid-induced acute kidney injury. Int Immunopharmacol (2021) 100:108142. doi: 10.1016/j.intimp.2021.108142 34555644

[44] Mittrucker HW Matsuyama T Grossman A Kundig TM Potter J Shahinian A. Requirement for the transcription factor LSIRF/IRF4 for mature b and T lymphocyte function. Science (1997) 275(5299):540–3. doi: 10.1126/science.275.5299.540 8999800

[45] Lassen S Lech M Rommele C Mittruecker HW Mak TW Anders HJ. Ischemia reperfusion induces IFN regulatory factor 4 in renal dendritic cells, which suppresses postischemic inflammation and prevents acute renal failure. J Immunol (2010) 185(3):1976–83. doi: 10.4049/jimmunol.0904207 20601597

[46] Tanaka S Tanaka T Kawakami T Takano H Sugahara M Saito H. Vascular adhesion protein-1 enhances neutrophil infiltration by generation of hydrogen peroxide in renal ischemia/reperfusion injury. Kidney Int (2017) 92(1):154–64. doi: 10.1016/j.kint.2017.01.014 28318627

[47] Li HY Lin HA Nien FJ Wu VC Jiang YD Chang TJ. Serum vascular adhesion protein-1 predicts end-stage renal disease in patients with type 2 diabetes. PloS One (2016) 11(2):e0147981. doi: 10.1371/journal.pone.0147981 26845338 PMC4742057

[48] Hoefman S Snelder N van Noort M Garcia-Hernandez A Onkels H Larsson TE. Mechanism-based modeling of the effect of a novel inhibitor of vascular adhesion protein-1 on albuminuria and renal function markers in patients with diabetic kidney disease. J Pharmacokinet Pharmacodyn (2021) 48(1):21–38. doi: 10.1007/s10928-020-09716-x 32929612 PMC7979602

[49] de Zeeuw D Renfurm RW Bakris G Rossing P Perkovic V Hou FF. Efficacy of a novel inhibitor of vascular adhesion protein-1 in reducing albuminuria in patients with diabetic kidney disease (ALBUM): A randomised, placebo-controlled, phase 2 trial. Lancet Diabetes Endocrinol (2018) 6(12):925–33. doi: 10.1016/S2213-8587(18)30289-4 30413396

[50] Day AJ Milner CM. TSG-6: A multifunctional protein with anti-inflammatory and tissue-protective properties. Matrix Biol (2019) 78-79:60–83. doi: 10.1016/j.matbio.2018.01.011 29362135

[51] Lee RH Pulin AA Seo MJ Kota DJ Ylostalo J Larson BL. Intravenous hMSCs improve myocardial infarction in mice because cells embolized in lung are activated to secrete the anti-inflammatory protein TSG-6. Cell Stem Cell (2009) 5(1):54–63. doi: 10.1016/j.stem.2009.05.003 19570514 PMC4154377

[52] Xu J Zheng J Zhu J. [Tumor necrosis factor-alpha induced protein 6 attenuates acute kidney injury following paraquat poisoning in rats]. Zhonghua Wei Zhong Bing Ji Jiu Yi Xue (2014) 26(6):405–8. doi: 10.3760/cma.j.issn.2095-4352.2014.06.008 24912639

[53] Cox SN Chiurlia S Divella C Rossini M Serino G Bonomini M. Formalin-fixed paraffin-embedded renal biopsy tissues: An underexploited biospecimen resource for gene expression profiling in IgA nephropathy. Sci Rep (2020) 10(1):15164. doi: 10.1038/s41598-020-72026-2 32938960 PMC7494931

