# Supplementary material for: Identification and validation of hub genes in drug induced acute kidney injury basing on integrated transcriptomic analysis
Source: Front Immunol. 2023 Mar 29;14:1126348. doi: 10.3389/fimmu.2023.1126348 (PMC10090697; doi:10.3389/fimmu.2023.1126348)
Supplement: Supplementary Figure 1 — The line diagram indicates first ten Hub genes results obtained by BottleNeck algorithm in Cytohubba plugin. [file DataSheet_1.pdf]

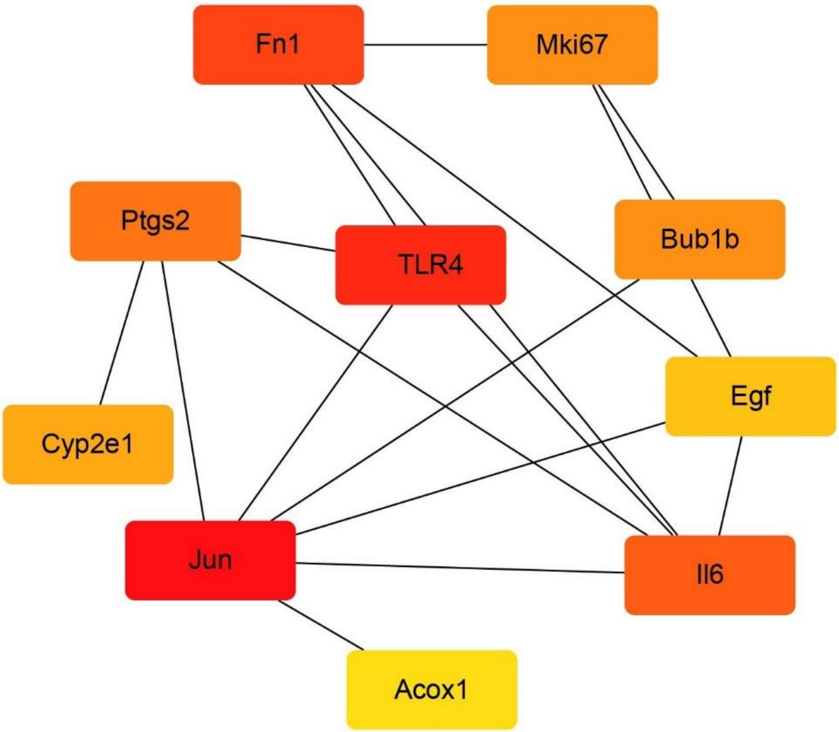

Figure S1. | The line diagram indicates first ten Hub genes results obtained by BottleNeck algorithm in Cytohubba plugin.

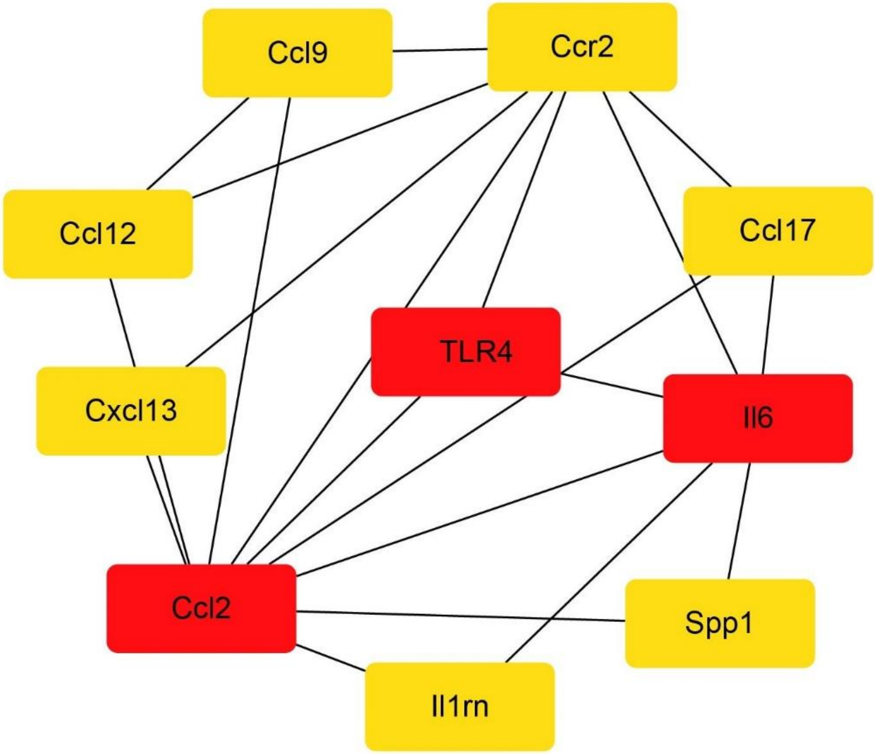

Figure S2. | The line diagram indicates first ten Hub genes results obtained by EPC algorithm in Cytohubba plugin.

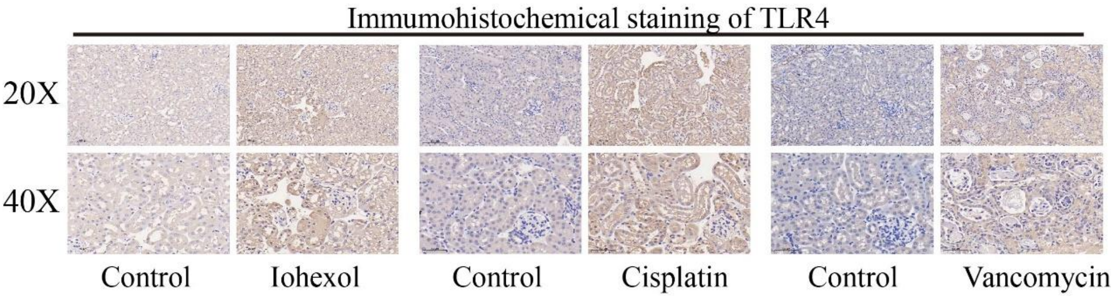

Figure S3. | Representative image of TLR4 immunohistochemistry in iohexol, cisplatin and vancomycin induced-AKI mice.
